# Spatiotemporal dynamics in the early stages of the 2009 A/H1N1 influenza pandemic

**DOI:** 10.1371/currents.RRN1026

**Published:** 2009-08-31

**Authors:** Thibaut Jombart, Rosalind M Eggo, Pete Dodd, Francois Balloux

**Affiliations:** ^*^Faculty of Medicine, Imperial College London; ^†^MRC Centre for Outbreak Analysis and Modelling, Imperial College London.; ^‡^Imperial College London and ^§^MRC Centre for Outbreak Analysis and Modelling, Dept of Infectious Disease Epidemiology, Imperial College, London, UK

## Abstract

Epidemiology and public health planning will increasingly rely on the analysis of genetic sequence data. The ongoing influenza A/H1N1 pandemic may represent a tipping point in this trend, with A/H1N1 being the first human pathogen routinely genotyped from the beginning of its spread. To take full advantage of this genetic information, we introduce a novel method to reconstruct the spatiotemporal dynamics of outbreaks from sequence data. The approach is based on a new paradigm were ancestries are inferred directly rather than through the reconstruction of most recent common ancestors (MRCAs) as in phylogenetics. Using 279 A/H1N1 hemagglutinin (HA) sequences, we confirm the emergence of the 2009 flu pandemic in Mexico. The virus initially spread to the US, and then to the rest of the world with both Mexico and the US acting as the main sources. While compatible with current epidemiological understanding of the 2009 H1N1 pandemic, our results provide a much finer picture of the spatiotemporal dynamics. The results also highlight how much additional epidemiological information can be gathered from genetic monitoring of a disease outbreak.

## Introduction

As the first pandemic of the 21^st^ century, the 2009 influenza A/H1N1 outbreak acts as a timely reminder of the looming threat posed by emerging infectious diseases. Indeed, the worldwide spread of a new deadly pathogen with sustained human-to-human transmission is one of the most worrying public health scenarios. Early containment based on robust scientific evidence is recognised as our best hope to avert a catastrophic situation if faced with the global spread of a deadly pathogen [1] [2] [3]. To be most effective, prophylactic interventions must be implemented early on and will be based on only very preliminary scientific evidence. Thus, it is crucial that all sources of useful information be considered. Genetic sequence data can now be generated essentially in real time and the analysis of sequence data has already been added to the toolbox of epidemiology and pandemic planning [4] [5] [6]. However, the statistical methodologies are currently lacking to harness the full potential of genetic sequence information. In particular there is to date no available method for reconstructing the spatiotemporal dynamics of a set of strains collected during an outbreak.

Current state of the art genetic methods for the reconstruction of pathogen genealogies rely on the phylogenetic paradigm [7] [8] [9] [10] based on the inference of putative most recent common ancestors (MRCAs) between pairs of sequences. The analysis of sequence data collected during disease outbreaks poses considerable challenges. Phylogenetic reconstruction is hampered by the limited genetic diversity inherent to the early stages of an outbreak as obtaining statistical support behind MRCAs requires extensive genetic polymorphism. More fundamentally, the reconstruction of MRCAs becomes inappropriate when ancestors and their descendents are both present in the sample analyzed, as is bound to happen during the early stages of an outbreak. To circumvent these problems, we introduce a new methodological paradigm for the analysis of genetic data structured in space and time (Fig. 1). Rather than reconstructing hypothetical MRCAs, we developed an algorithm called *SeqTrack*, which directly reconstructs the most likely ancestries in space and time. 

We applied the novel methodology to 297 hemagglutinin (HA) sequences of the 2009 H1N1 pandemic influenza virus [11] [12]. This allowed us to reconstruct the spread of this newly emerged pathogen in considerable detail. 

**Figure:**
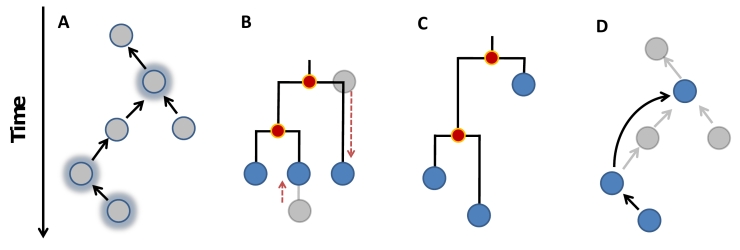
**Figure 1. Illustration of possible reconstructions of genealogical relations.** Panel a represents a hypothetical genealogy of five strains from which three were sampled (highlighted by a blue glow). Panel b and c illustrate phylogenetic reconstructions where the red dots represent inferred nodes; panel b corresponds to a classical phylogenetic reconstruction and c to a tree that accounts for heterochronous sampling (e.g. Beast software [8]). Panel d illustrates the direct ancestry reconstruction method implemented in *SeqTrack*.  While none of the methods recovers the correct genealogy (panels b-d), the best reconstruction is provided by *SeqTrack* in this example (panel d).

## Methods

### SeqTrack algorithm

Our method aims at uncovering ancestries between strains of an outbreak using their genotype and collection date. The fundamental innovation of our approach is to seek ancestors directly from the sampled strains, rather than attempting to reconstruct unobserved and hypothetical ancestral genotypes. Because ancestries are in essence temporally-oriented connections between strains, it seemed natural to tackle the problem of inferring genealogies within graph theory.

*SeqTrack* relies on three fundamental, yet simple observations. First, each observed strain has one, and only one ancestor. Second, ancestors always precede their descendents in time. And third, among all possible ancestries of a given strain, some are more likely than others, and this likelihood can be inferred from the amount of genetic differentiation between the strains considered. The purpose of *SeqTrack* is to identify the most likely genealogy. Technically, this problem translates into finding the optimum branching in a directed graph, where each node is a strain, and where a given strain is connected to all strains occurring strictly later.

Let \begin{equation*}\mathcal{G} = (S,E,w)\end{equation*} be a directed, weighted graph where \begin{equation*}S=\left\{ s_1, \ldots, s_n \right\}\end{equation*}  is the set of vertices corresponding to the \begin{equation*}n\end{equation*} sampled strains, with associated collection dates \begin{equation*}T=\left\{ t_1, \ldots, t_n \right\}\end{equation*}. \begin{equation*}E\end{equation*}  is the set of directed edges of \begin{equation*}\mathcal{G}\end{equation*} modelling all possible ancestries in \begin{equation*}S\end{equation*} , such that \begin{equation*}(s_i, s_j) \in E\end{equation*}  if and only if \begin{equation*}t_i < t_j\end{equation*}. The weight function \begin{equation*}w: E \rightarrow \mathbb{R}\end{equation*}  assigns a weight to each possible ancestry, which can reflect the genetic similarity or dissimilarity between the considered pairs of strains. For instance, \begin{equation*}w\end{equation*}could be defined as a genetic distance or similarity, or as the log-likelihood resulting from a probabilistic model of evolution. The weight of a subset \begin{equation*}A\end{equation*} of \begin{equation*}E\end{equation*} is computed as \begin{equation*}w_A = \sum_{e \in A} w(e)\end{equation*}. The ‘best’ genealogy of the sampled strains is the directed spanning tree (*i.e.*, ‘branching’ reaching all the nodes) \begin{equation*}\mathcal{J} = (S,B,w)\end{equation*} with \begin{equation*}B \subseteq E\end{equation*}  optimizing (*i.e.*, minimizing or maximizing) \begin{equation*}w_B\end{equation*}. 

This problem has been solved by Edmonds/Chu-Liu [13] [14], who developed an algorithm to find \begin{equation*}\mathcal{J}\end{equation*} so that \begin{equation*}w_B\end{equation*}  is maximum or minimum. The algorithm proceeds by identifying optimum ancestors for each node at the exception of the root (the oldest strain), and then recursively removes potential cycles. However, in our case, cycles are impossible as ancestries cannot go back in time, which greatly simplifies computations. 

The entire *SeqTrack* procedure has been implemented in the *adegenet* package [15] for the R software [16]. This implementation allows specifying any weight and choosing the type of optimization (minimization or maximization of total weight) to take advantage of the method’s versatility. In this study, we considered that ancestries involving the fewest mutational steps were the most likely. The chosen weights were therefore simply the number of mutations separating two strains. In the case of an influenza outbreak, this parsimony approach is justified by the low amount of genetic differentiation between emerging flu strains, and the absence of recombination and reverse mutations. The resulting ancestry path connects all the sampled strains while minimizing the required number of mutational steps, and respecting their temporal ordering. Mapping these results allows visualization of the inferred spatiotemporal spread of the new influenza virus.

For biological interpretation, it can be desirable to measure the reliability of some specific segments of the obtained optimum genealogy. The support of inferred ancestries can be assessed by computing the likelihood of the number of mutations (\begin{equation*}\nu _k\end{equation*}, with \begin{equation*}k=1,\ldots,n\end{equation*}) observed between the two strains of the edge \begin{equation*}b_k\end{equation*}. This probability depends on the length (\begin{equation*}L\end{equation*}, in number of nucleotides) and mutation rate (μ, in number of mutations per nucleotides and per day) of the considered DNA sequences, and on the period of time elapsed (\begin{equation*}\Delta _t\end{equation*}, in days) between the two strains’ collection dates. It is then simply derived from the probability mass function of a Binomial distribution with probability μ  and \begin{equation*}L\Delta_t\end{equation*} trials:

\begin{equation*}P(\nu _k | \mu, L, \Delta_t) = \frac{(L\Delta_t)!}{\nu_k! (L\Delta_t - \nu_k)!}\mu^{\nu_k}(1-\mu)^{L\Delta_t - \nu_k}\end{equation*}

This likelihood can be represented on all edges using a color coding to allow a better assessment of the results (Fig. 2).

### Sequence data

Genetic data were used to infer the spatiotemporal spread of the swine-derived influenza A/H1N1 pandemic. We analyzed all full-length HA segments available from Genbank ([17], http://www.ncbi.nlm.nih.gov/Genbank/index.html) as of the 26/06/2009. DNA sequences and their annotations (including sampling dates and locations) were retrieved and processed using *ad hoc* scripts in the R language [16]. Only strains for which collection date and location were available were retained. Duplicates existed for some strains due to different passages, sometimes resulting in slightly different sequences for the same strain. In these cases, significantly shorter sequences were discarded, as well as sequences obtained from amplification in eggs since this method is known to induce additional ‘artificial’ mutations [18]. 

The alignment of all retained sequences was realised using Clustalw [19] and refined by hand using Jalview [20]. The final alignment contained 279 sequences of 1668 nucleotides, with collection dates ranging from 30/03/2009 to 18/06/2009. Raw pairwise genetic distances (in number of differing nucleotides) were computed using the *ape *package [21] for the R software.

Flight data was used to assess the role played by air traffic in the dispersal of the pathogen. Passenger flows between countries were compiled from a list of all commercial flights that occurred between 5th May 2008 and 4th April 2009, purchased from OAG (http://www.oag.com/). Numbers of passengers between countries were then correlated to the number of inferred transmissions, and tested using the usual Student *t*-test.

### Simulated data

Individual-based simulations were used to further assess the power of our method to uncover ancestries from outbreak genetic data. All simulations were performed in the R software, using procedures newly implemented in the *adegenet* package. Datasets were obtained by simulating genealogies of haplotypes evolving stochastically in time and space. 

Two types of simulations, differing in the process governing the spatial spread of the strains, were performed. The first scheme allowed strains to disperse on a 5-by-5 regular grid according to a random Poisson process, with identical probabilities to move in all directions. This scheme is later referred to as ‘random diffusion’. The second type of simulation used a stochastic dispersal based on pre-defined probabilities of new strains to migrate from one location to another. This allowed us to recreate the effect of ‘sources’ and ‘sinks’, *i.e.* some locations seeding many other locations, including very distant ones, and other locations attracting migration but not seeding other places, respectively. This type of simulation is later referred to as ‘structured dispersal’.

Because the number of strains grows exponentially in outbreak simulations, the number of haplotypes that need to be handled rapidly becomes intractable. This issue can be solved by randomly discarding a sufficient number of strains at each generation, so that the number of strains never exceeds a given threshold. The advantage of this approach is that the average pairwise genetic differentiation between strains should not differ statistically between the sample and the population. 

Another limiting factor for simulating outbreaks relates to the number of simulated generations. For instance, swine-derived influenza A/H1N1 virus typically has a short generation time (1.91 days, IC_95%_=[1.30-2.71]; [22]), which would make realistic simulation time-consuming. As an alternative, we simulated haplotypes on fewer generations (10 on average), using a larger mutation rate. The average level of genetic differentiation among simulated strains was of the same order of magnitude as that of the analyzed influenza sequences.

For each simulation scheme (random diffusion and structured dispersal), ten datasets were simulated, each of which gave rise to ten samples of 300 haplotypes. 

## Results

To evaluate the performance of the *SeqTrack* algorithm we analyzed simulated data. Outbreak data were generated using two different spatial models.  In the first set of simulations, we assumed a random uniform diffusion of strains in space. These simulations were complemented by a second set of simulations run under structured dispersal, where strains had variable probabilities of seeding different locations. This allowed for a source and sink dynamics more representative of actual outbreaks. Analysis of the simulations indicated that the true ancestral haplotype (100% identity) was successfully retrieved in 77% of cases on average throughout all simulations (CI_95%_=[77%-78%]; Fig. 2). The correct location was inferred more frequently under source and sink dynamics than under homogeneous dispersal (79% and 56%, respectively; t=-22.11, df=237, p<2e-16; Fig. 2). 

**Figure:**
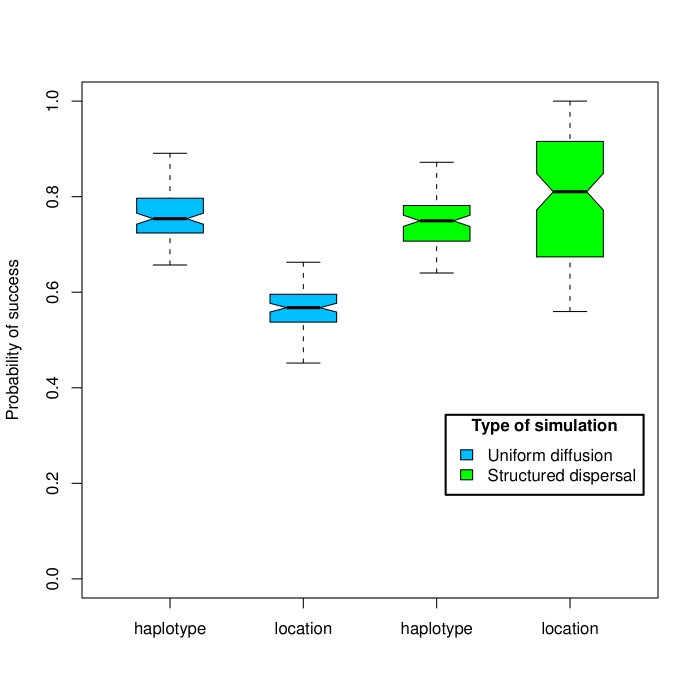
**Figure 2. Results from simulation data. **The proportion of correct assignments for haplotypes and locations are represented as boxplots.

Having tested that the method satisfactorily assigned the correct ancestral locations on simulated data, we applied the *SeqTrack* algorithm to a dataset of 279 near-complete hemagglutinin (HA) sequences collected between the 3^rd^ of March and the 18^th^ of June 2009. While no sequences are available for the first confirmed cases (La Gloria, 15^th^ February; [22]), the time window defined by the collection dates extends from the early stages of the outbreak to the global pandemic, which was officially declared by the WHO on the 11th June 2009. In figure 2, we represent the reconstruction by our method of the spatiotemporal dynamics of the 2009 H1N1 pandemic split into three major stages: (i) initial spread in Mexico and the US (Fig. 3a), (ii) sustained transmission within North America and spread to the rest of the world (Fig. 3b) and (iii) further worldwide spread and secondary outbreaks outside the Americas (Fig. 3c).

**Figure:**
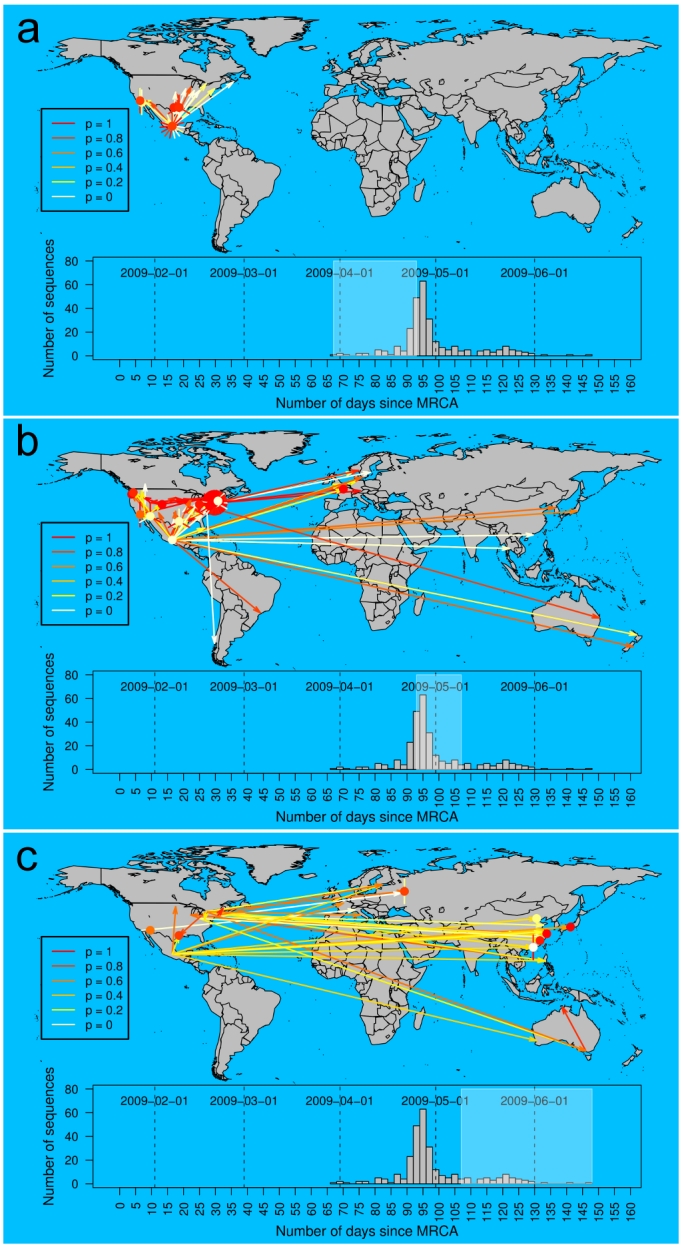
**Figure 3. Maps of inferred ancestries of the swine-origin A/H1N1 flu pandemic, based on 279**

**hemagglutinin (HA) DNA sequences available on Genbank as of the 26/06/2009.** Arrows represent inferred ancestries, with colors indicating the statistical support (Equation 2). Local transmissions are shown as dots for single events, and sunflowers for multiple transmissions. The inset histogram displays the number of strains analyzed by collection date. Dates are provided as days since the most recent common ancestor (MRCA), estimated as the 21/01/2009 [22]. The three different panels display successive stages of the pandemic (corresponding time frame highlighted in the inset): a) transmissions from Mexico to the US, b) transmissions within the US, and beginning of world-wide spread, and c) further worldwide spread, likely from unobserved outbreaks outside the Americas.

The initial stage ending around the 20^th^ of April is characterized by transmissions from Mexico to the US as well as some local transmissions both within Mexico and the US (Fig. 3a). This pattern gives further support to the A/H1N1 having originated in Mexico. Secondary outbreaks within the US become widespread during the second phase while Mexico and the US also start seeding the rest of the world (Fig. 3b). It is also during this stage that we infer the first case of a within-country transmission outside the Americas (France on the 1^st^ of May). Many of the ancestries over large geographic distances are well supported, suggesting that the intercontinental inferred ancestries accurately capture the actual pattern of transmission from the Americas to Europe, Asia, and Australia. Conversely, the fraction of transmissions with low associated statistical support may represent cases where intermediate stages in the ancestry path have not been sampled. This is also likely to be the case during the third phase, where many transmissions originating from Mexico, the US, and Canada are characterized by faint statistical support. Conversely, the increasing number of local transmissions in Asia, Russia and Australia are characterised by high statistical support and point to the emergence of multiple secondary outbreaks. 

Flight traffic is widely recognized as an important determinant for the spread of seasonal and pandemic influenza at large geographic scales [1] [2] [3] [23] [24]. Thus, we evaluated the relationship between passenger-flow and the number of inferred transmissions between countries. Both quantities were fairly correlated (*r* = 0.54; *t*-test: *t *= 3.75; df = 34; P = 3.3 x 10^-4^), but this correlation was largely driven by the high connectivity of the US with Mexico and Canada. A far more remarkable aspect of the spatiotemporal reconstruction of the 2009 A/H1N1 pandemic is the near-absence of implausible transmission events (Fig. 3). The only possible exceptions to this pattern are two Mexican sequences which trace their ancestry back to the US. However, the statistical support was very low in both cases with likelihoods of 2.1x10-7 and 0.11. The inference of at least one implausible event from the US to Mexico was inescapable as the oldest sequences present in the dataset were all sampled in the US. The absence of implausible transmissions during the later stages of the pandemic is particularly surprising as we expected *SeqTrack* to struggle with the increasingly sparse sampling of the extant viral strains. Over the time period analyzed, the available samples become less representative of the global viral population; A/H1N1 cases have been increasing essentially exponentially but the sequencing effort has diminished from the end of April. 

## Discussion

We have introduced a new methodological paradigm for the analysis of genetic data sampled during an outbreak. We developed a method rooted in this paradigm which behaves satisfactorily on simulated data and reconstructs a highly plausible global scenario for the early stages of the 2009 A/H1N1 pandemic. We reconstruct an initial outbreak in Mexico, and a rapid spread to the US. At a later stage, Mexico and the US act as the principal source for the worldwide diffusion of the virus, with secondary foci of infection becoming increasingly common with time outside the Americas. However, there is considerable potential for methodological developments and improvements of the *SeqTraq *algorithm, both in the short and the long term. 

In this paper we used maximum parsimony because the origin of A/H1N1 is sufficiently recent for homoplasies (recombination or back mutations) to be safely ignored. However, *SeqTrack* genealogies could be optimized on other criteria, such as genetic distances or log-likelihoods based on complex models of sequence evolution (see Methods). Another development of immediate interest relates to the use of diverse genetic information. All analyzes were conducted on the hemagglutinin (HA) segment. The influenza genome comprises eight segments each harbouring one gene. As the A/H1N1 sequencing effort was undertaken independently by a large number of labs, the available genetic information is extremely heterogeneous. Sequences deposited on Genbank for various strains range from small fragments of specific genes to complete genomes, including a majority of strains for which an essentially random combination of segments were sequenced. This translates in a considerable loss of information. *S*uch heterogeneous information could be accommodated by averaging genetic differentiation across segments, using appropriate weights to account for different substitution rates and segment lengths. 

However, including a large fraction of strains with heterogeneous sequencing coverage would require accurate estimates of substitution rates, which can be difficult to obtain for emerging pathogens. Another possible concern is that different segments may exhibit incongruent genealogies due to different selective pressures, reassortments and/or intra-genomic interactions [4] [25] [26] [27] [28]. Some other developments would require more effort. It would in particular be desirable to consider the various parameters of the model from a probabilistic perspective. For instance, the date of probable transmission could be better captured by a distribution of the time during which a specific sequence remains unaltered rather than a fixed collection date. Moreover, a probabilistic framework would also allow incorporating information besides genetic sequences and collection dates, such as prevalence data or spatial connectivity between locations. 

The 2009 A/H1N1 influenza pandemic is the fourth in modern history. 20^th^ century pandemics occurred in 1918, 1957 and 1968. In all three previous cases, the newly emerged strain replaced the strain that was circulating and causing seasonal (winter) epidemics. The current seasonal influenza  is a descendent of the 1968 H3N2 pandemic and has been co-circulating with a less virulent H1N1 strain at lower frequencies since 1977 [4]. Whether the 2009 A/H1N1 will fully displace the current seasonal influenza strains remains to be seen.  Seasonal influenza strains have strongly cyclical dynamics, with the majority of cases observed during the winter in both hemispheres. The epidemics in the Northern and Southern hemisphere are believed to be essentially independent but both are seeded most years from Southeast Asia which acts as a reservoir for flu [4] [5]. The factors underlying seasonal dynamics are poorly understood despite decades of research [29]. There is considerable disagreement over the importance of climatic variables such as temperature and humidity in driving the seasonal patterns worldwide [30] [31]. An additional confounding effect arises from school holidays, which are also believed to be important drivers of seasonality in influenza [32] [33]. 

In principle, our method would be ideal to shed further light on the effect of human travel, climate and additional factors such as timing of school holidays on the underlying factors behind the spread of the 2009 A/H1N1 pandemic. However, the main difficulty stems from the current limitation of the available data. The ratio between the number of confirmed and actual cases is likely to vary greatly between countries. Moreover, the number of strains that have been sequenced for each country is uncorrelated with the number of confirmed cases. This leads to considerable difficulties for disentangling the main factors behind the spread of the 2009 A/H1N1 pandemic. What is certain is that human travel must be important. The majority of long distance movement is likely to be mediated by airline travel both for seasonal and pandemic flu [1] [2] [3] [23] [25] [34]. Despite the limitations of the genetic dataset, we observe a highly significant correlation between flight traffic and the routes inferred by *SeqTrack*. At this stage, we are unable to provide a quantitative estimate of the effect of climate. However, the sustained spread of the 2009 A/H1N1 strain in the Northern hemisphere during the spring and summer suggests it may be less sensitive to climatic conditions than the seasonal influenza strains currently in circulation.

The *SeqTrack* algorithm has been specifically developed to model the spatiotemporal dynamics of the 2009 A/H1N1 pandemic. However, the approach should be applicable to essentially any genetic data collected during the early stages of an outbreak. This could include localised outbreaks within a school or a community but also epidemics at much larger geographical scale. As the method does not require extensive genetic polymorphism it should prove effective in bacteria and fungi, in addition to viruses. A straightforward application would be the reconstruction of the global spread of the fungus *Batrachochytrium dendrobatidis*, which is driving amphibian populations to extinction worldwide [35]. We hope that the performance of the method, together with its wide applicability, flexibility, computational speed and free availability within the *adegenet* package [15] will convince infectious disease epidemiologists to adopt it as an integral part of the toolkit for disease outbreak analysis. More generally, we hope that the novel paradigm it is based upon will open a whole new field in the statistical genetics of emerging pathogens. 

## Acknowledgments

We are most grateful to all our colleagues who sequenced strains from the 2009 influenza A/H1N1 pandemic and made them publicly available. We feel particularly indebted towards Nancy Cox, Mark Greenway, Naomi Komadina, Andreas Nitsche, John Pasick, Tomas Pumarola and Pilaipan Puthavathana for their willingness to provide additional information on sequences they submitted on Genbank. We address many thanks to Vicente Acuña for providing insights about the graph theory, and to William Hanage for commenting on the earlier version of the work.

## Funding information

FB acknowledges financial support from the BBSRC and the MRC.

## Competing interests

The authors have declared that no competing interests exist.
